# Patient–healthcare provider communication and age-related hearing loss: a qualitative study of patients’ perspectives

**DOI:** 10.1007/s11845-023-03432-4

**Published:** 2023-06-29

**Authors:** Lorita Lee Mei Lu, Patrick Henn, Colm O’Tuathaigh, Simon Smith

**Affiliations:** 1 School of Medicine, University College Cork, Cork, Ireland; 2 Application of Science to Simulation Based Education and Research (ASSERT) Centre, School of Medicine, University College Cork, Cork, Ireland; 3 Medical Education Unit, School of Medicine, University College Cork, Cork, Ireland

**Keywords:** Age-related hearing loss, Clinical interaction, Communication, Presbycusis

## Abstract

**Background:**

The prevalence of age-related hearing loss (ARHL) significantly increases in people aged 60 and older. Medical errors are frequently reported because of communication breakdown, especially for patients with ARHL.

**Aims:**

This qualitative study focuses on identifying the communication challenges faced by people aged over 65 with ARHL and potential ameliorative strategies based on the participants’ personal experiences.

**Methods:**

Thirteen participants, attending a support service for older adults with hearing loss in the South of Ireland, were recruited using convenience sampling. Semi-structured interviews were conducted with participants. Interviews were audio-recorded and transcribed using NVivo 12 software. Braun and Clarke’s thematic analysis methodology was used to identify themes arising from two main study domains: difficulties faced during the most recent healthcare interaction and suggestions for improving overall healthcare communication.

**Results:**

Older adults with hearing loss identified general mishearing, lack of awareness and use of medical terminology to be the cause of ineffective communication. Raising awareness of the impact of presbycusis on clinical interaction among healthcare professionals was cited as being of crucial importance. Other helpful strategies include repeat and rephrase, use of written information, providing context, minimizing ambient noise, continuity of care, longer consultation length and good body language.

**Conclusion:**

Effective clinical communication can be achieved through a clear understanding of the patient’s perspective. Healthcare providers should be made aware of the hearing issues and associated communication difficulties posed, within the context of the development of patient-centred strategies to improve patient safety.

## Introduction

Interpersonal communication has been described as a critical tool for life adjustment, linking people with their environment [1]. Communication challenges are commonly reported by older people, either due to normal ageing or communication disorders related to various conditions [2]. Despite the cause, communicating well with older adults remains a significant challenge for many healthcare providers and is often complicated by sensory impairments and/or cognitive problems [2]. Previous studies have also identified hearing loss as a modificable risk factor for cognitive decline [3, 4]. According to the National Institute on Deafness and Other Communication Disorders in the USA, hearing loss is ranked as the third most prevalent chronic condition in older adults [4]. Age-related hearing loss (ARHL), also known as presbycusis, is the second most common illness in aged people worldwide [5]. It affects approximately one-third of people aged 65 to 74 and almost half of those over the age of 75 [6]. This presents a significant challenge in delivering healthcare, as the number of older adults continues to grow.

Presbycusis is sensorineural, meaning the primary damage happens in the hair-like cells within the cochlea or the hearing nerve. It is characterized by decreased hearing sensitivity, especially for high-frequency sound, and most often affects both ears. Previous quantitative studies have confirmed that presbycusis has a negative impact on clinical communication, across both hospital and primary care clinical settings [2]. People with this type of hearing loss tend to exhibit difficulties with speech perception and comprehension because of associated difficulties with high-frequency consonants, which are fundamental for word discrimination, such as distinguishing between words such as “time” and “dime” [7]. These difficulties are exacerbated in a noisy environment and slowly progresses to loss of hearing sensitivity at lower frequency sound, which makes it harder to understand words in a quieter setting. This results in a loss of clarity of speech sounds; increasing the volume of the speech may or may not improve the condition.

Older adults with specific communication needs are significantly more likely to experience preventable adverse events and functional decline in hospital [8]. This can be challenging during an inpatient stay and may limit a person’s confidence to participate in their care and their ability to follow instructions [9]. Lower ratings of patient–physician communication and overall healthcare have been reported among older adults with self-reported hearing loss [10]. In most instances, patients would feel embarrassed and frustrated having to ask the others to repeat words and sentences, which ultimately causes them to withdraw from social activities [11]. Due to poorly adapted communication strategies, it is reported that people with hearing loss perceive their social skills as poor. Consequently, the combination of hearing impairment and a poor coping strategy contributes to poor self-esteem in these patients [12].

In 2001, the Institute of Medicine highlighted the importance of effective communication in facilitating knowledge transfer and shared decision-making involved in patient-centred care [13]. A recent study found that only 44% of older adults using multiple medications have spoken to a healthcare provider about possible drug interactions, suggesting an important gap in communication [14]. This was exacerbated by the observations that older adults often receive prescriptions from multiple providers who are not all using a shared electronic health record system [14]. Even more concerning, 30% only partially understood the healthcare provider’s explanation for the requirement for a medication, tests or procedure, and 10% did not understand it at all [15]. Thus, it is no surprise that patients who are deaf or hard of hearing are at high risk of breakdowns in healthcare communication, which is the leading cause of medical errors. The present study aims to explore the experiences of patients with ARHL in interacting with healthcare providers, including perceived communication challenges as well as, importantly, their views on how such communication problems can be addressed. Through learning about patients’ experiences of clinical communication, we aimed to suggest improvements to how patient–healthcare practitioner interaction can be delivered.

## Methods

### Study design

A qualitative study, employing one-to-one semi-structured interviews, was conducted during October 2019. The one-to-one format, as opposed to group interviews or focus groups, was chosen as a method of collecting data, as it enables participants with presbycusis the freedom and comfort to express their views in regard to patient–healthcare communication, without the challenge of communicating in group settings [16].

### Participants

All older adults who attended the “Hard of Hearing” support services for older adults with hearing loss provided by the Cork Deaf Association were invited to participate in the semi-structured one-on-one interview with the following inclusion criteria: participants aged 65 and above, with age-related hearing impairment and attending services provided by the Cork Deaf Association. The Cork Deaf Association is a local charitable organization that is part-funded by the Irish Health Service Executive. The selected age group was chosen based on the known fact that presbycusis has the highest incidence among the over-60 s [17]. The Hard of Hearing support group provides opportunities for older adults with hearing loss to participate in social outings, receive access to information talks, attend coffee mornings etc.

### Data collection

**Fig. 1:**
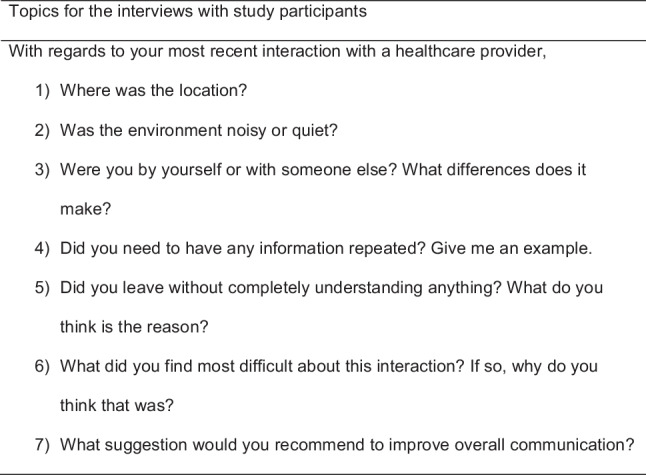
Interview topic guide

Thirteen participants were recruited using convenience sampling. Informed consent was obtained before conducting the semi-structured 1-to-1 interview. All interviews were conducted at the office of the Cork Deaf Association. Each interview lasted between 10 and 15 min. Participants were asked to complete a demographic profile form at the beginning of each interview session. The sessions were audio-recorded. The moderator of the interview chaired the sessions with a topic guide. The topic guide consisted of seven questions asking participants to share their thoughts regarding (i) difficulties faced during the most recent interaction and (ii) suggestions for improving overall communication. Figure 1 provides a summary of the interview topics. Participants were advised to approach the researchers or supporting staff of participating organizations if they experienced any distress arising from study participation.

### Research instruments and data analysis

Audio recordings were transcribed, and the final verbatim was analysed using Braun and Clarke’s thematic analysis method [18]. Thematic analysis is a flexible and distinctive systematic method that has been used to explore patients’ experiences of healthcare services through identifying, analysing and organizing qualitative data. In this approach, data was read and re-read several times by two researchers (COT, LLML) to become familiar with the data (step 1). Individual interview transcripts were uploaded onto NVivo 12, which was used for generating initial codes (step 2) and developing initial themes (step 3). A single response could be coded to more than one theme. Two researchers (COT, LLML) were involved in revising themes (step 4) and determining and designating themes (step 5). Lastly, two major domains and their respective themes were generated before researchers worked together in yielding the final report (step 6) [18]. All the quotes were condensed for clarity.

### Ethical considerations and data privacy

Ethical approval was obtained from the Social Research Ethics Committee (SREC) of the School of Medicine, University College Cork. Written informed consent was obtained from the participants before the study commenced. Participants were designated a study identity to anonymize all personal data.

## Results

### Participant characteristics

Table 1 provides an overview of participants’ socio-demographic and hearing loss characteristics, respectively. The mean (± SD) age was 75.1 ± 6.20 years; 53.8% were male and 46.2% were female. 92.3% had bilateral hearing loss; 30% of the 76.9% acquired hearing loss participants were exposed to a previous noisy work environment. All reported using hearing aids.

**Table 1:** Baseline socio-demographic and hearing loss characteristics

**Variables**	**Mean (SD)**	***n*** **(%)**
**Demographics**		
Age	75.08 (6.198)	
**Gender**		
Male Female		7 (53.8) 6 (46.2)
**Types of hearing loss**		
Unilateral Bilateral		1 (7.69) 12 (92.3)
**Causes of hearing loss**		
Congenital Acquired Both		0 (0.0) 10 (76.9) 3 (23.1)
**Previous noisy work environment**		
Yes No		4 (30.8) 9 (69.2)
**Hearing aids/amplifier use**		
Yes No		13 (100.0) 0 (0.0)

*SD* standard deviation, *n* = frequency

### Overview of themes

We identified themes arising from questions related to both study domains. The first domain “Difficulties faced during the most recent healthcare interaction” explored the challenges participants faced in a clinical setting. They often misheard the conversation which can be attributed to healthcare providers’ lack of understanding of their communication needs and their use of medical terminology. The second domain “Suggestions for improving healthcare communication” relates to participants’ opinions regarding patient-led solutions to promoting a better quality of care. This includes repeating back information, displaying positive non-verbal cues and enhancing awareness of communication needs of older adults.

### Domain 1: Difficulties faced during the most recent healthcare interaction

#### Theme 1: General mishearing

Several participants mentioned background noise as the primary cause of hearing difficulty in a hospital setting. On the other hand, consultation in the GP offices is regarded as almost always pleasant and quiet, as they attributed this experience to the privilege of having a private room that enables one-to-one interaction:

I think that on one-to-one, it’s very easy. But when there’s a group around, it’s difficult. For hearing people, it’s difficult with all the noise. I say that like in a meeting, like a noisy ward. (P13, female).

Furthermore, participants noted the impact of foreign accents on comprehension. Despite their attempts to understand accents, some people have more difficulty understanding speakers with strong and different accents. It is an unavoidable phenomenon, particularly as it relates to healthcare providers for whom English is not their native language:

A foreign doctor, I mean they speak the best, they can but I’m lost. (P9, female).

It is not uncommon that fast-paced speech can make it unclear for the other party to grasp the meaning in a conversation. This occurs if the person is a naturally fast talker, and it can exacerbate in the presence of nervousness, urgency and mental fatigue. Lack of verbal pauses provides no time for patients, particularly those with hearing impairment, to comprehend the content and concepts.

If the person talks too fast, I find it hard to understand. (P4, male).

#### Theme 2: Lack of awareness

It was suggested that people generally equate presbycusis with total deafness despite it being a gradual loss of hearing that occurs in most people as they grow older. Participants reported frustration with having to constantly explain the slight yet crucial difference between these forms of hearing loss. Participants reported that as they have already experienced such situations numerous times, it is not uncommon for a trained healthcare professional to be confused too:

I put it down to people who were not aware of what it’s like to have hard of hearing. There is a perception ‘oh, you’re deaf’, full stop. But there are two sections – deafness and hard of hearing. Hard of hearing is the problem. You go into any doctor but there’s nothing about hearing, even the family doctors are not aware of it. (P1, male).

Despite being well informed regarding the issue of hard of hearing, participants revealed that they often felt that little to no accommodation has been provided to maximize the patients’ benefits during the consultation. This is reflected in various disruptions including constant interruptions and the absence of a loop system:

One time I just had to say to them that ‘I have hard of hearing now’. I said ‘call my name loud’ or whatever like that, but they don’t like to be told that. (P2, male).

Some participants also highlighted the impact of insufficient consultation time as they require extra time to fully understand and process the conversation compared to a normal hearing individual:

They don’t give enough time for you to explain. (P4, male).

#### Theme 3: Use of medical jargon

The common consultation content misinterpreted by patients with hearing loss related to medication and the use of medical terminology:

Information about medication, mostly. (P4, male).

See, if you go to a clinic now and you’re talking and they might call in the other person and they are talking too. They are staying there and I don’t have a clue what they are talking about. (P9, female).

### Domain 2: Suggestions for improving healthcare communication

#### Theme 1: Repeating information

One way to upgrade patient–healthcare provider communication is by focusing on promoting patient comprehension of health information. This can be achieved through clear communication, confirming understanding, asking questions and gaining clarity. Verbal confirmation is cited as often vital to gauge the patient’s understanding at the end of the consultation:

Because he was aware of my hearing problem and he said to me a couple of times now ‘tell me if I’m not getting through’. (P1, male).

In most cases, written notes are required to help patients better retain medical information. It is notably useful in instances such as information overload, patients with memory impairment, coverage of complex topics and where there is emphasis on certain details. One of the participants revealed the limitation of verbal confirmation as it is dependent on the receiver’s capacity to process and retain data:

There’ll be also stages where I know no matter how many times the person repeats it, I won’t hear it. They have to write it down. (P10, male).

Some participants acknowledged the challenge of coping with changing subjects amid discussion, yet some find it more troubling. They admitted getting lost in the conversation when the topic is unwillingly changed. Focus on one topic at a time is ideally the best option when dealing with an older adult with hearing impairment:

I am speaking about something and it’s grand, I can understand. I can follow points then let’s say there’s a third party comes in and they communicate, they changed the context, I’m lost. (P10, male).

#### Theme 2: Raise awareness

Healthcare providers should be made aware of the hearing issue and the difficulties posed by it. Continuity of care with the same doctor is cited as the easiest and straightforward way to address this challenge:

I think that’s important to continue care with the same doctor, that’s important. (P3, female).

Also, the likelihood of getting a longer consultation duration is higher with the same doctor who is aware of the patient’s hearing problem as compared to those who are not. They are presumably more attentive to patients’ needs without rushing through the consultation. Most participants noted that extra time is very much needed even with new doctors since they generally need the information to be repeated or vice versa:

You know because they have to repeat themselves which takes up that bit of time. (P9, female).

Participants’ perceptions towards attending a consultation with company were also explored. Surprisingly, every one of the participants preferred to be alone. Reasons relating to personal preferences and convenience were then disclosed, yet the most striking one being self-autonomy. They elaborated it as a sense of empowerment and confidence without feeling like an “ill person” in the absence of another person:

He has come up with me in the past but I found it better when I present my own on a one to one because the audiologist and whoever is with my son tends to, they don’t realize that I suppose, they tend to exclude you and they talk to one another as if you are a thing, you know, that has to be discussed …. So, I was better one to one with the audiologist myself. (P3, female).

Notification may be the key to raising awareness. Just like any known drug allergy, participants recommended labelling “hearing impairment” on top of their medical records. This will bring immediate attention to the doctor and appropriate adjustments can be made before seeing the patient:

The doctor knows because on my thing on the computer is ‘deaf’, profoundly deaf or something so he knows. I think once he looks at my thing and sees ‘deaf’ on top, they take that into account. (P3, female).

#### Theme 3: Non-verbal cues

Great emphasis was placed on maintaining eye contact when it comes to effective communication. The power of good eye contact in the context of patient–healthcare provider relationship includes but is not limited to building a strong rapport and trust. It makes them feel listened to which in turn leads to the willingness to share their problem, promoting better patient engagement and medication adherence:

As long as they look at me, as long as they don’t talk to me with their face down and they do sometimes forget and they do that, you know, or sometimes they move over to get something and they talk with their back to me. Now I have to be facing the person. Eye contact definitely. (P3, female).

One of the participants mentioned that facing the other person when talking would make lip reading much easier:

Whereas I will be completely dependent on lip reading so I have to see its full face. (P10, male).

Another participant indicated that communication errors can be reduced if doctors position themselves nearer to the patients:

I have to sit very close to listen to the consultant. (P1, male).

Participants reportedly observe subtle body language to quickly “read between the lines” and interpret the meaning behind the silent clues. The display of positive body language is therefore highly encouraged in every setting:

I often think two people could say the same thing. You could say something to me now, some very offhand, you know, hurtful. On the other hand, you could use the same words to me but it’s how the context and that, I would think a lot of body language. (P5, female).

## Discussion

This study aimed to understand perceptions of older adults with hearing loss regarding patient–healthcare practitioner interactions and how they might be improved. Nearly half of older adults, including hearing aids users, reported mishearing healthcare providers in clinical settings [19]. Studies concluded that background noise, multiple people talking at the same time and poor pronunciation between two similar words are barriers to achieving clear communication [11, 19]. When background noise is present, consonant confusion can easily give rise to communication misunderstanding. Many consonants contain high-frequency sounds, which are often lost, while low-frequency sounds remain full and clear. This explains why participants typically report mishearing the content despite being able to hear the speaker’s voice. The speech becomes unclear, and people talking appears mumbled. Their clarity of speech comprehension has diminished as they are missing a large portion of important speech signals [20]. Fortunately, participants report fewer problems when visiting their general practitioner (GP) due to its communication-friendly environment (i.e. a quiet, well-lit room with furniture arranged for face-to-face interactions) [21].

Ageism has been cited as an important barrier to good communication between older patients and healthcare providers [22]. These participants highlighted the lack of understanding and adaptation from their healthcare providers who are either primary care doctors or internists in their respective medical fields. Unlike clinicians involved in geriatric care, who are often more educated and sensitive to the unique healthcare needs of older adults, they are more likely to engage in overaccommodation known as elderspeak — addressing the elderly in an overly simple and patronizing way. Overaccommodation occurs when the speaker is over-reliant on negative stereotypes of ageing [23]. Often, the first instinct when facing older people with hearing loss is to increase the volume of the speech by talking loudly or shouting, which has a paradoxical effect in those with presbycusis, as it raises the sound pitch. It is more beneficial to slow down the rate of speech which in turn improves articulation and clarity. Indeed, it has been reported that individuals with hearing loss suffer important communication problems if the speaker fails to articulate slowly or deliberately [24]. Additionally, mild and moderate accents can affect a patient’s ability in recognizing monosyllabic and multisyllabic words [25].

Currently, within primary care, the minimum consultation length recommended is 10 min. However, a survey of the British Medical Association found that 92% of 15,560 GPs perceived that 10 min was inadequate for primary care consultations [26]. This is especially true given that GPs are increasingly dealing with a growing elderly population with chronic and complex conditions. Patients believe that insufficient consultation time results in poorer quality of care, a higher chance of needing a prescription or attending more frequently [27]. In particular, some participants revealed feeling rushed and not able to get their point across without “clock watching” behaviour exhibited by some GPs. Thus, alternatives such as telephone calls and email consultations should be offered according to individual clinical needs. A longer consultation can then be provided for those with special needs, including patients with presbycusis, as they require visual cues and assistive technology when communicating [28].

Medical jargon and medication are the two most misinterpreted consultation content as pointed out by the participants. A false understanding of commonly used medical terms can jeopardize patient–healthcare provider communication and decision-making [10, 29].

Diagnosis, treatment and prognosis are commonly misinterpreted by patients which include misunderstandings around medical information due to complicated medication dose and regimen [11, 30]. It is therefore the prescriber’s responsibility to inquire about the patient’s understanding of medical information to prevent detrimental drug adverse events. Given the importance of this matter, surprisingly, few studies exist that guide healthcare providers on how to approach this task. One study [31] offers some insight into three types of approach: yes–No, tell back–directive and tell back–collaborative. The yes–no approach focused on close-ended questions, whereas the tell back–directive method used open-ended questions that were physician-centred. The tell back–collaborative is a patient-centred open-ended approach, making it clear that it is a shared responsibility between the patient and practitioner. Patients showed a significant preference for the tell back–collaborative as they view the request for tell back as evidence of practitioners’ care and concern for them personally. In short, it is critical to invite the patients to restate their understanding of the information in their own words within a shame-free environment.

Unsurprisingly, participants wish to continue receiving care from the same doctors who are familiar with their condition. Relationship continuity has been shown to increase security and trust within the patient–doctor relationship, which in turn increases patient’s willingness to accept medical advice and adherence to long-term preventive regimens [31]. Despite participants favoured attending their consultation alone, Adelman et al. [32] revealed the importance of having a frequent third-party present when it comes to decision-making and conduit for education. The third-party may play the role of advocate, passive participant or antagonist. However, it can sometimes feel awkward for the practitioner to have an additional person in the room acting as an interpreter [33]. Nevertheless, it is crucial for all parties involved to respect the patient’s autonomy in any circumstances.

Interestingly, many sounds can be seen that are hard to hear. Lip reading significantly improved speech discrimination in older adults with hearing loss; it was undoubtedly greater in individuals with presbycusis [34]. A relevant study highlighted the use of visual cues in recognizing voice sounds as the production of each phoneme is associated with a particular facial expression pattern [11]. These visual hints can be hidden if the speaker is looking away while taking notes on the computer or addresses the recipient while performing another task as highlighted by one of the participants. This explains why some participants repeatedly position themselves in front of their healthcare providers to allow a better view of lip reading. Moreover, maintaining eye contact is just as important as keeping the mouth and face visible when interacting with any patient. It shows attentiveness and interest in what is being said. Eye contact has been positively linked to patient’s assessment of clinician empathy and rating of attributes, for instance, connectedness and how much they liked the clinician [35]. Participants agreed that eye contact opens up communication and helps build rapport and trust.

The qualitative study allows broad, open-ended inquiry which encourages participants to raise issues that matter most to them and helps the researchers to determine the idiographic causes. It is restricted to older adults attending a specialist service, which might suggest recruitment bias. The results of this study may lack generalization because the sample was restricted to participants who were available to proceed with the interview at a specific date and time. Hence, they are not necessarily representative of the population of interest due to the small sample size and context specificity. Additionally, the presence and degree of hearing loss, as well as hearing aid use, were based on self-report rather than objective data, excluding the possibility to explore the relationship between the level of hearing loss and difficulties faced during the clinical encounter. The use of hearing aids during clinical consultations was also not explored during the study interview.

## Conclusion

Effective communication assists health professionals in identifying individual needs in a holistic approach. Key targets for intervention arising from this study include putting an alert or code on the patient record to ensure that the healthcare provider knows that this patient has certain communication needs due to their hearing loss. From recognizing the challenge of hard-of-hearing patients to making adjustments such as rephrase and assess for understanding, good body language, face-to-face orientation, communication-friendly environment, written information and adaptable consultation length, these measures will improve patient outcomes and lead to greater patient–healthcare provider satisfaction.

## Data Availability

The data that support the findings of this study are available from the corresponding author upon reasonable request.

## Code availability

The data coding results from this study are available from the corresponding author upon reasonable request.
